# THEORIZING FORTITUDE: MINDFULNESS

**DOI:** 10.1093/geroni/igae098.1983

**Published:** 2024-12-31

**Authors:** Kristoffer Rhoads

**Affiliations:** University of Washington School of Medicine, Seattle, Washington, United States

## Abstract

Normal and pathological aging both require a tremendous amount of resilience and fortitude. Successful aging also requires the ability to fully and accurately appreciate one’s past, rectified in the context of one’s present and future. Defined as the practice of intentionally cultivating non-judgmental awareness of the present moment, without criticism or evaluation, mindfulness practices offer a powerful tool to enhance acceptance of the “here and now” while cultivating compassion for the aging self and others. This presentation will briefly review the tenets and principles of mindfulness meditation as conceptualized implemented by Jon Kabat-Zinn, PhD and colleagues at the Center for Mindfulness in Medicine, Health Care, and Society at the University of Massachusetts Medical School. Specifically, this presentation will explore applications to older adults with and without later life neurological disorders. This will include benefits of accepting one’s current abilities and strengths decoupled from comparisons to earlier life as well as disengaging from a catastrophic narrative of future losses. The presentation will also review the benefits of mindfulness practices for older adults living with memory loss and dementia, including how mindfulness meditation practices may interact with neurobiological underpinnings and risk factor modification of conditions such as Alzheimer’s disease, vascular dementia, depression and anxiety.

